# Inhibitory effects of *Calocybe indica *macrofungi on experimental benign prostatic hyperplasia in rats

**DOI:** 10.22038/IJBMS.2022.64972.14309

**Published:** 2023-01

**Authors:** Remigius I. Onoja, John I. Ihedioha, Shodeinde VO. Shoyinka, Arinze S. Ezema, Nnenna T. Emejuo, Anthony C. Mgbeahuruike, Benjamin I. Emesiani, Wilson Obidah, Iyi Clinton

**Affiliations:** 1 Department of Veterinary Pathology and Microbiology, Faculty of Veterinary Medicine, University of Nigeria, 410001, Nsukka, Nigeria; 2 Institute for Drug Herbal Medicine Excipients Research and Development, Department of Pharmaceutical Microbiology and Biotechnology, Faculty of Pharmaceutical Sciences, University of Nigeria, Nsukka, Nigeria; 3 Department of Biochemistry, School of Life Sciences, Modibbo Adama University of Technology Yola, Yola, Nigeria

**Keywords:** Agaricales, *Calocybe indica*, Hyperplasia, Pathology, Prostate-specific antigen, Testosterone

## Abstract

**Objective(s):**

This study was designed to investigate the protective effects of *Calocybe indica* extract on testosterone-induced benign prostatic hyperplasia in rats.

**Materials and Methods:**

In this study, 60 adult Sprague Dawley rats were randomly divided into six equal groups, one group served as the normal control, five of the groups were administered subcutaneous testosterone propionate for 28 days to induce benign prostatic hyperplasia, three of the five groups were simultaneously administered three graded doses of *C. indica* extract while one group was administered finasteride as the standard drug and the other left as untreated BPH model group given testosterone propionate only. BPH in the prostate gland was detected through gross appearance, prostate weight, and biochemical and histopathological analyses.

**Results:**

Increased prostate weight, serum prostate-specific antigen (PSA), and epithelial thickness were observed in the untreated testosterone-induced BPH model. Administration of finasteride and *C. indica* extract led to a reduction in prostate weight, prostatic index, serum PSA, serum levels of testosterone, and prostatic epithelial thickness, and increased luminal diameter.

**Conclusion:**

Administration of *C. indica* extract suppressed the pathophysiological effects of benign prostatic hyperplasia in rats. Thus, *C. indica* mushroom is a potential pharmacological candidate for the management of BPH in man or dogs.

## Introduction

Benign prostatic hyperplasia (BPH) is an age-related, non-cancerous, benign, and endocrine-controlled enlargement of the prostate gland in man and dogs which leads to bladder outflow obstruction, lower urinary tract symptoms, sepsis, irreversible bladder damage, renal failure, or even death (1-6). BPH occurs in about two-thirds of un-neutered (intact) dogs as early as over 5 years of age and 95 % of intact dogs older than 9 years of age as reported in many countries (7-9). In men, it occurs mainly after 50 years of age, and the frequency increases with age (10-12). The etiology of BPH is not fully elucidated; however, the etio-pathologic mechanism seems to be endocrine controlled and involves alterations in the metabolism of androgens and estrogens in aging men and dogs (9, 13). Although diet, lifestyle, and oxidative stress are reported as some of the risk factors, BPH is known to be caused by the enzymatic conversion of testosterone into its more active metabolite dihydrotestosterone by 5-alpha reductase enzyme in the prostatic cells of men and dogs (14, 15). This enzyme is the target for drug therapy aimed at reducing the size of the prostate in BPH. Thus, the two main classes of conventional therapeutic agents used to manage BPH are the 5-alpha reductase inhibitors (5-ARIs) and the alpha-blockers (16, 17). Surgical procedures are also applied if medical therapy does not alleviate urinary symptoms. However, the conventional methods of BPH management have adverse side effects. The alpha-blockers cause dizziness, headache, weakness, asthenia, retrograde ejaculation, and nasal congestion, while the 5-alpha reductase inhibitors cause ejaculatory dysfunction, erectile dysfunction, gynecomastia, and also lower PSA by 50% after 6 months of therapy which affect prostate cancer diagnosis (18-20). Surgical interventions provide immediate improvement in alleviating BPH symptoms but have the highest risk of developing sexual dysfunction and urinary incontinence when compared with other therapeutic options (21, 22). It is because of these side effects that patients have turned to natural products for the management of BPH, as they are believed to have pharmacological properties such as anti-androgenic, antiproliferative, anti-oxidant, and anti-inflammatory properties (6, 23). Therefore, the number of cases requiring alternative medication is on the increase (24) as natural products show limited adverse effects compared with conventional medications (25). The macrofungi, *Calocybe indica* has been proven to have significant medicinal properties such as antioxidant, anticancer, immune stimulation, antidiabetic, and hepatoprotective effects due to its possession of bioactive compounds such as β-glucans and polyphenols (26-29). However, there is a paucity of information available in the scientific literature on the effects of *C. indica *on BPH*. *Thus, this study was designed to evaluate the possible protective effect of *C. indica *extract on experimental BPH in rats.

## Materials and Methods

***Drugs and chemicals***

Finasteride, the standard drug for the treatment of BPH, and testosterone propionate (TP) for the induction of BPH were purchased from Sigma Chemical Co (St Louis, Missouri, USA). A testosterone ELISA kit and a PSA ELISA kit were purchased from Elabscience**®** (Houston, Texas, USA). All other routine chemicals and reagents for the study were of analytical grade.

***Mushroom collection and extraction***

Fruiting bodies of the *C. indica* mushroom were purchased from a reputable commercial mushroom farm and identified by a plant biologist. The mushroom was dried under shade for 10 days and ground into powder. Five hundred grams (500 g) of the powdered material was soaked in 70% methanol with manual shaking at 2-hr intervals for 72 hr after which it was filtered through Whatman paper (No. 1) and concentrated using a rotary evaporator. The dried *C. indica* extract (CLE) was stored in a refrigerator at 4 °C until the time of use.

***Animals***

Sixty 10–12 weeks old healthy Sprague-Dawley outbred male albino rats (*Rattus norvegicus*) weighing between 160–180 g were used for this study. The rats were housed in cages of size 60 cm x 45 cm x45 cm with wood shavings as bedding and acclimatized at a temperature of 25±4 °C and relative humidity of 65±5% with an alternating 12 hr light and dark cycle for two weeks. They were fed rat chow and given water *ad libitum.* Ethical approval for this study was obtained from the Faculty of Veterinary Medicine Institutional Animal Care and Use Committee of our institution based on the experimental protocols as directed by the National Institute of Health Guide for Care and Use of Laboratory Animals (30). 

***Acute toxicity study of C. indica extracts***

The acute toxicity of *C. indica* extract was studied according to OECD guideline 423 for testing of chemicals (31). Adult male rats were administered 625, 1250, 2500, and 5000 mg/kg doses of the extract orally and observed for clinical signs of toxicity and mortality. No mortality or signs of toxicity were observed in animals administered 5000 mg/kg of the extract. Hence, the median lethal dose (LD_50_) was considered to be greater than 5000 mg/kg body weight in rats.

***Experimental design***

After a brief acclimation period of two weeks, the rats were randomly divided based on body weight into six equal groups of ten rats each as follows: (a) control, (b) TP 3 mg/kg only, (c) 3 mg/kg TP+ 5 mg/ kg finasteride, (d) 3 mg/kg TP+250 mg/kg CLE, (e) 3 mg/kg TP+ 500 mg/kg CLE, and (f) 3 mg/kg TP+1000 mg/kg CLE. The rats were induced with BPH through subcutaneous administration of testosterone propionate (TP) at 3 mg/kg body weight daily for four weeks as previously described (32) and simultaneously dosed with various extracts of the mushroom daily for four weeks. The effective dose of finasteride for the treatment of BPH and testosterone used to induce BPH was as previously recommended (33). The volume for oral administration of PBS, finasteride, and the extract was 5 ml/kg and 2 ml/kg for SSC injection of olive oil and TP, respectively (33). 

***Body and prostatic weights***

The body weight of the rats was determined on the day before drug administration and the day of sample collection, after which the animals were sacrificed and the prostate glands weighed. The prostatic index was determined using the formula: 

Prostatic index =Prostate weight × 100%Body weight 

***Sample collection***

At the end of the study, the rats were fasted overnight, weighed and about 4 ml of blood samples were collected from the retro-orbital plexus of each rat into plain sample bottles in order to obtain serum for biochemical study before they were humanely sacrificed. The prostate glands were removed immediately and weighed before they were fixed in a 10% neutral formalin solution for histopathology.

***Assay for testosterone***

ELISA kits were used for the quantitative determination of testosterone concentration according to the manufacturer’s instructions. Briefly, 2 µl aliquots of standards and samples were dispensed into their respective wells in ready-to-use microtitre plates pre-coated with anti-hormone IgG antibodies. After addition of 100 µl anti-hormone HRP conjugate (1:100 dilutions) to each well, the plates were incubated for 30 min at room temperature. The contents of the well were then aspirated and the wells were washed three times with 200 µl of wash solution. The enzyme reaction was started by addition of the chromogen (tetramethylbenzidine/hydrogen peroxide system) into each well. Plates were then incubated for 10 min. The reaction was stopped by addition of 100 µl of 0.15 M H_2_SO_4_. Absorbance was measured at 450 nm.

***Assay for prostate-specific antigen (PSA)***

This was estimated using a competitive enzyme immunoassay technique performed by an ELISA method according to the kit manufacturer’s instruction (Elabscience**®**, Houston, Texas, USA). The micro ELISA plate provided in the kit had been pre-coated with an antibody specific to Rat PSA. Standards or samples were added to the micro ELISA plate wells and combined with the specific antibody. Then a biotinylated detection antibody specific for Rat PSA and Avidin-Horseradish Peroxidase (HRP) conjugate was added successively to each microplate well and incubated. Free components were washed away. The substrate solution was added to each well. Only those wells that contain Rat PSA, biotinylated detection antibody, and Avidin-HRP conjugate appeared blue in color. The enzyme-substrate reaction was terminated by the addition of a stop solution and the color turned yellow. The optical density (OD) was measured spectrophotometrically at a wavelength of 450 nm ± 2 nm. The concentrations of Rat PSA in the samples, which is proportional to the OD value, were calculated by comparing the OD values of the samples with the standard curve. 

***Histopathological evaluation***

The ventral prostate lobes of the prostate glands were manually processed for histopathological studies after fixing in 10% neutral buffered formalin (NBF) for 48 hr. The samples were then dehydrated in ascending grades of ethanol gradient (70%, 80%, and 90%) for 1 hr 30 min each, 100% absolute ethanol for 1 hr 30 min (two changes) before they were cleared in chloroform overnight, followed by infiltration with paraffin in a bath at 60 °C for 1 hr 30 min (two changes), after which they were embedded in paraffin. Five-micrometer thick sections of the samples were cut using a rotary microtome (Shandon, Finesse 325, ThermoFisher Scientific, Luton, England). The sections were then floated in a water bath at a temperature of 40 °C to spread before they were mounted on glass slides coated with egg albumin. Thereafter, sections were deparaffinized in xylene (two changes) for 15 min each and rehydrated through descending grades of ethanol (100%, 90%, 80%, and 70%) for 10 min each and then changed to distilled water for 15 min. Sections were manually stained for 15 min in Harris hematoxylin (HHS16, Millipore Sigma,), washed in tap water to ‘blue’ the nuclei, and counterstained in Alcoholic Eosin Y (515, 3,801,615; Leica Biosystems Inc, Buffalo Grove, IL, USA) for 5 min., dehydrated through an ascending ethanol gradient, cleared in xylene, and coverslipped using DPX mountant (C06522, Sigma-Aldrich St. Louis, Missouri, USA) for light microscopy (34)**.** Photomicrographs of the sections were captured using Motic Images plus 2.0 digital cameras (Motic China Group Ltd 1999–2004). 

***Histochemistry***

The cut sections were deparaffinized and rehydrated as described earlier. The sections were oxidized in 1% periodic acid for 10 min and then placed in Schiff’s reagent (Sigma-Aldrich, St Louis, MO, USA) for 15 min. The sections were then passed through warm running water for 10 min to wash off the Schiff’s reagent. A solution (0.02%) of fast green FCF (C.I. 42053, Sigma-Aldrich St. Louis, Missouri, USA) was used to counter-stain the sections for 30 sec. Tissue sections were rinsed quickly in distilled water and dehydrated through an ascending ethanol gradient, cleared in xylene, and coverslipped using DPX mountant (C06522, Sigma-Aldrich St. Louis, Missouri, USA) for light microscopy (34) using a Periodic acid–Schiff (PAS) stained kidney section as control. Photomicrographs of the sections were captured using Motic Images plus 2.0 digital cameras (Motic China Group Ltd 1999–2004). 

***Statistical analysis***

Statistical analysis of the data obtained was carried out using SPSS software, version 23. Comparisons were performed using one-way ANOVA followed by post-hoc test. Data was presented as mean ± standard error of the mean (S.E.M).Values with *P*<0.05 was accepted as significant.

## Results

***Body weight changes, prostatic weight, and prostatic index***

**Figure 1:**
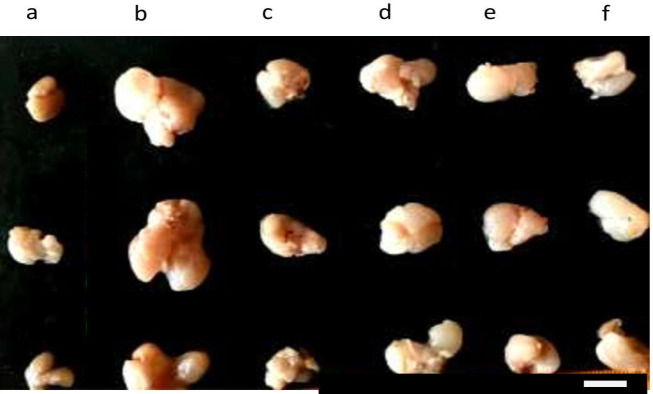
Gross pictures of the prostate of BPH rats treated with graded doses of CLE: (a) control, (b) TP 3 mg/kg only, (c) 3 mg/kg TP+ 5 mg/ kg finasteride, (d) 3 mg/kg TP+250 mg/kg CLE, (e) 3 mg/kg TP+ 500 mg/kg CLE, and (f) 3 mg/kg TP+1000 mg/kg CLE. (Scale bar = 10 mm)

BPH: Benign prostatic hyperplasia; CLE: *Calocybe indica* extract; TP: Testosterone propionate

Grossly, the size of the prostate gland of the untreated testosterone-induced BPH group was larger compared with the control group. Treatment with finasteride and CLE led to a considerable reduction in the size of the prostate gland in BPH-induced groups of rats as shown in Figure 1. The mean body weights of the experimental groups were not significantly (*P*<0.05) different from each other, but, the mean body weight of the untreated testosterone-induced BPH group was higher than that of the control, finasteride, and CLE groups after 28 days of treatment, as treatment with both finasteride and the three different doses of CLE led to a lower mean body weight as shown in Table 1. The mean prostate weight of the untreated testosterone-induced BPH group was significantly (*P*<0.05) higher than the control, finasteride, and CLE-treated groups. However, the mean prostatic weight of rats treated with the three different doses of CLE was (*P*<0.05) significantly higher than that of the control but lower than the finasteride-treated group. The mean prostatic index of the untreated testosterone-induced BPH group was significantly (*P*<0.05) higher than the control, finasteride, and CLE-treated groups, but the mean prostatic index of rats treated with the three different doses of CLE were (*P*<0.05) significantly higher than the control but lower than the finasteride-treated group (Table 1).

***Serum testosterone concentration***

**Figure 2:**
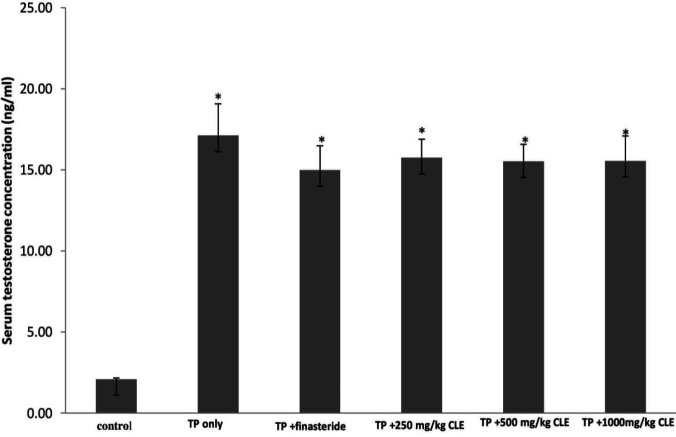
Effects of graded doses of CLE on mean serum testosterone concentration of the experimental groups: (a) control, (b) TP 3 mg/kg only, (c) 3 mg/kg TP+ 5 mg/ kg finasteride, (d) 3 mg/kg TP+250 mg/kg CLE, (e) 3 mg/kg TP+ 500 mg/kg CLE, and (f) 3 mg/kg TP+1000 mg/kg CLE. The values are expressed as mean ± SEM (n=5). A value of *P*<0.05 was considered to be statistically significant. Significance was determined by one-way ANOVA followed by* post hoc* test, *P<0.05, significantly different from the negative control

TP: Testosterone propionate; CLE: *Calocybe indica* extract

The mean serum testosterone levels of the untreated testosterone-induced BPH group were significantly (*P*<0.05) higher than that of the control. Although the mean serum testosterone levels of the testosterone model (BPH) group were higher than the values in all the CLE-treated groups, this was not significant (*P*>0.05). There was no significant (*P*>0.05) difference in the mean serum testosterone levels between the finasteride-treated group C and the CLE treated groups (Figure 2).

***Prostate-specific antigen (PSA) concentration***

**Figure 3:**
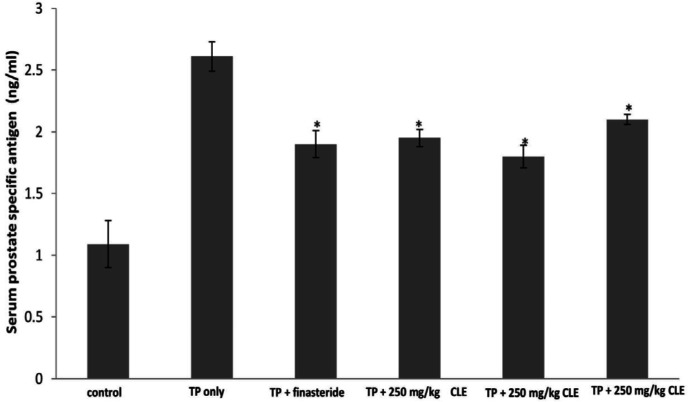
Effects of graded doses of CLE on mean serum PSA concentration of the experimental groups: (a) control, (b) TP 3 mg/kg only, (c) 3 mg/kg TP+ 5 mg/ kg finasteride, (d) 3 mg/kg TP+250 mg/kg CLE, (e) 3 mg/kg TP+ 500 mg/kg CLE, and (f) 3 mg/kg TP+1000 mg/kg CLE. The values are expressed as mean ± SEM (n=5). A value of *P*<0.05 was considered to be statistically significant. Significance was determined by one-way ANOVA followed by a *post hoc* test, *P<0.05, significantly different from the group administered TP only

CLE: *Calocybe indica* extract; PAS: Periodic acid–Schiff; TP: Testosterone propionate

The PSA level of the untreated testosterone-induced BPH group was significantly (*P*<0.05) higher than the normal control group while the finasteride-treated group showed a significant (*P*<0.05) decrease in PSA level compared with the untreated testosterone-induced BPH group. The administration of the various doses of CLE also led to a significant (*P*<0.05) decrease in the PSA level of the BPH rats compared with the untreated testosterone-induced BPH group as shown in Figure 3.

***Histopathology***

**Figure 4:**
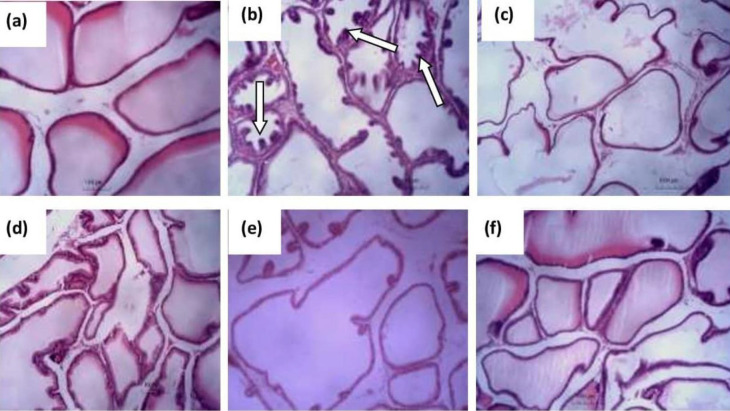
Representative photomicrographs of the cross-section of the prostate of rats administered graded doses of CLE: (a) control, (b) TP 3 mg/kg only, (c) 3 mg/kg TP+ 5 mg/ kg finasteride, (d) 3 mg/kg TP+250 mg/kg CLE, (e) 3 mg/kg TP+ 500 mg/kg CLE, and (f) 3 mg/kg TP+1000 mg/kg CLE. See the areas of epithelial projections into the lumen (arrows) in b compared with a, c, d, e, and f. H & E stain.100x

TP: Testosterone propionate; CLE: *Calocybe indica* extract

The histological features of the prostate gland from control rats showed normal histological architecture characterized by tubules of irregular diameter and lumen. The acini were separated by a fibromuscular stroma, blood vessels, and lymphatics (Figure 4a). However, the untreated testosterone-induced BPH model group showed hyperplastic glandular epithelium with projections into the lumen leading to a reduction in the luminal diameter of the acini of the glands compared with the control (Figure 4b). There was also an increase in the level of eosinophilic secretions in the lumen compared with the control group. The histology of the prostate of the finasteride-treated group showed a reduction in epithelial cell projections into the lumen (Figure 4c), just as treatment with the various doses of the CLE had a dose-dependent restoration of normal glandular structures with mild epithelial projections into the lumen (Figure 4d-f).

***Histochemistry***

**Figure 5:**
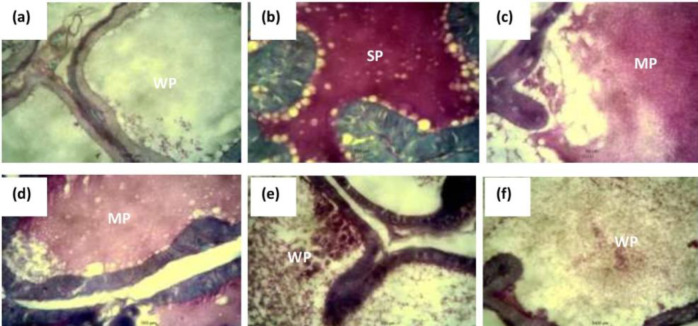
Representative photomicrographs of the cross-section of PAS + fast green counterstaining of the prostate of BPH rats administered graded doses of CLE: (a) control, (b) TP 3 mg/kg only, (c) 3 mg/kg TP+ 5 mg/ kg finasteride, (d) 3 mg/kg TP+250 mg/kg CLE, (e) 3 mg/kg TP+ 500 mg/kg CLE, and (f) 3 mg/kg TP+1000 mg/kg CLE. Note the strong PAS positivity of luminal secretions in b (SP) which has a moderate reaction in c and d (MP) compared with the weak reaction in a, e, and f (WP). PAS stain.400 x

PAS: Periodic acid–Schiff; BPH: Benign prostatic hyperplasia; CLE: *Calocybe indica* extract

There was a strong positivity for neutral mucin in the intra-luminal secretions of the prostate in the untreated testosterone-induced BPH model group compared with the control group after histochemical staining with PAS (Figure 5c). However, there was a mild positivity for neutral mucins in the luminal secretions of the prostate after treatment with finasteride and various doses of the CLE as shown in Figure 5c-f.

**Table 1:** Effects of graded doses of CLE on mean body weight and prostate weight of testosterone-induced BPH rats

Parameter groups	_Initial bwt (g)_	_Final bwt (g)_	_Prostate wt (g) _	_Prostate index (%)_
a(negative control)	184.67±1.33	229.00±6.56	0.27±0.03^a^	0.12±0.02^a^
b(TP 3 mg/kg only)	184.67±2.03	252.00±8.74	1.20±0.13^b^	0.48±0.06^b^
c(3 mg/kg TP+ 5 mg/ kg finasteride	184.33±1.86	230.00±11.14	0.46±0.10^ad^	0.21±0.05^ac^
d(3 mg/kg TP+250 mg/kg CLE	184.00±1.15	248.00±6.08	0.67±0.02^dc^	0.27±0.01^c^
e(3 mg/kg TP+ 500 mg/kg CLE	184.67±0.88	235.67±7.14	0.76±0.11^c^	0.32±0.06^c^
f(3 mg/kg TP+1000 mg/kg CLE	184.33±0.88	239.00±7.00	0.73±0.02^c^	0.30±0.01^c^

a b c Different alphabetical superscripts in a column indicate significant differences between the means of the groups, *P*<0.05

g: Grams; mg: Milligram; k: kilogram; TP: Testosterone propionate; BWt: Body weight; CLE: *Calocybe indica* extract; BPH: Benign prostatic hyperplasia

## Discussion

A number of studies have investigated the effects of natural products on BPH in which, similar to this study, daily doses of testosterone propionate for about 4 weeks were administered to induce the disease (24, 35, 36). In the present testosterone-induced BPH study, a rat model was used, which is very well established. The effects of testosterone on prostatic growth in rats have previously been documented and used to assess the effects of drugs used for prostatic hyperplasia treatment, including saw palmetto fruit lipid extract (35, 37, 38). There was a gross increase in size associated with a significant increase in prostate weight and prostatic index in the untreated BPH group compared with the normal control group. This has been attributed to the growth in the epithelial cell numbers in the prostate tissue which is increased in BPH and serves as a marker of the TP-induced BPH model and in natural disease cases in man or animals (36, 39, 40). This shows that BPH was properly induced in this experimental model. It is known that when prostate enlargement occurs, the urethral canal will be constricted and this can lead to partial or sometimes complete obstruction of the canal (41). Administration of finasteride or *C. indica* extract showed a decrease in prostatic weights and prostatic index of BPH rats suggesting that finasteride or *C. indica* extract has the potential of reducing the size of the prostate gland. There are several reports that the bioactive compounds present in *C. indica* extract are responsible for amelioration of lots of diseases, and that *C. indica* extract shows potent efficacy in different disease models in animals. The serum testosterone concentration of untreated testosterone-induced BPH rats was significantly higher compared with the normal control group. This may be due to the exogenous hormone administration. Treatment with finasteride or *C. indica* extract led to a non-significant reduction in serum testosterone concentration of finasteride and *C. indica* extract compared with the untreated testosterone-induced BPH rats but was significantly higher than the control. This could be due to the 5α-reductase inhibitory activity of both finasteride and *C. indica* extract which may have prevented the conversion of testosterone to dihydrotestosterone (DHT). The progression of BPH is androgen dependent. Thus, testosterone seems to act as a prohormone that is metabolized to its most active form, DHT, in the prostate through the enzymatic action of 5α-reductase. DHT is responsible for the pathogenesis and progression of BPH (9, 13**)**. Thus, the results of this study suggest that *C. indica* extract may reduce the expression of 5α-R related to the cellular proliferation associated with BPH. Prostate-specific antigen (PSA) is a protein produced by the cells of the prostate gland and it is used as a diagnostic biomarker in BPH and other prostatic diseases such as prostate cancer or chronic prostatitis (35, 37, 38). It is correlated with prostate size and when a decrease in PSA levels is observed in serum, it is assumed that the test substance in question is having therapeutic effects on the conditions of the prostate. In the present study, there was a high serum concentration of PSA in the sera of untreated testosterone-induced BPH rats compared with the control, which agrees with previous reports as an indication of hyperplasia. This reduced considerably after treatment with finasteride *and C. indica* extract, which is also an indication of its 5a-reductase activity, suggesting that the extracts could alleviate the expression of prostate biomarkers (PSA) in BPH. Histopathological observations in the prostate of the untreated testosterone-induced BPH rats which revealed proliferative changes like increased height of the columnar epithelia, multifocal epithelial thickening, and folding of the lining epithelia into the glandular lumen are typical of BPH and are consistent with previous reports (42). These changes were severe in the untreated testosterone-induced BPH rats but were mild following treatment with finasteride* and C. indica* extract. It has been reported that in BPH, there is increased glandular secretion into the lumen (43, 44). The intense expression of glandular secretions as seen under the PAS stained sections in the untreated testosterone-induced BPH rats was reduced after treatment with finasteride and* C. indica* extract. According to previous research, various mushrooms, including *Ganoderma lucidum, Phellius linteus*, and *Paecilomyces tenuipes* were effective in treating BPH (45-47). Most mushrooms contain a large amount of various physiologically active substances, such as β-glucan, polyphenol, and flavonoids, which have been reported to inhibit the activity of 5AR2 and attenuate the promotion of BPH (45-47). The results of this study show that *C. indica* extract *has* inhibitory effects on the cellular changes of the prostate and other pathophysiological changes associated with the testosterone-induced model of BPH. 

## Conclusion

The present study was designed to determine the effects of *C. indica* on histopathological and biochemical markers of benign prostatic hyperplasia in rats. The results showed that this mushroom could inhibit and moderate the histopathological and biochemical alterations associated with BPH in rats. This implies that *C. indica* extract has the potential to serve as a new pharmacological candidate or therapeutic option for the treatment of benign prostatic hyperplasia.

## Authors’ Contributins

RIO, JII, and SVO Conceived and designed the experiments; RIO, ASE, NTE, and IC Performed the experiments; WO, JII, and RIO Analyzed the data; BIE and ACM Contributed reagents/ materials/analysis tools; and RIO, JII, and SVO Wrote the article. All authors agree to be accountable for all aspects of the work, ensuring integrity and accuracy. The authors declare that all data were generated in-house and that no paper mill was used. 

## Conflicts of Interest

The authors declare no conflicts of interest.
